# Ageism, ableism, and their associated factors among international undergraduate medical students in China: a cross-sectional study

**DOI:** 10.3389/fpubh.2026.1864380

**Published:** 2026-08-20

**Authors:** Azmat Ullah, Hongwei Qi, Atif Rashid, Yutong Pan, Zakaria Zaheen, Qi Li, Yuting Song

**Affiliations:** 1 School of Nursing, Qingdao University, Qingdao, Shandong, China; 2 School of Computer Science and Technology, Qingdao University, Qingdao, Shandong, China

**Keywords:** ableism, ageism, healthcare education, internalized bias, Relational Aging Anxiety Scale, undergraduate medical students

## Abstract

**Background:**

Ageism and ableism are common attitudes that have significant effects on the quality of healthcare provided to older adults and people with impairments. Despite growing concern, research into these biases among undergraduate medical students is limited, particularly in Asian educational contexts. As future healthcare providers, medical students’ early views on aging and disability must be addressed; however, little research has investigated the interrelationship between ageism and ableism in this population.

**Methods:**

A cross-sectional survey was conducted among 199 undergraduate medical students at Qingdao University in China. Data were gathered using the Relational Aging Anxiety Scale, which has five subscales: affinity for older adults, internalized ageism, relational ageism, internalized ableism, and relational ableism. All subscales showed Variable internal consistency (0.429–0.861), with three subscales meeting acceptable thresholds and two falling below the conventional 0.70 criterion. Pearson correlation analysis and four multiple linear regression models were used to investigate Factors associated with ageism and ableism outcomes, with demographic variables (age, gender, year of study, and prior experience) and attitudinal subscales serving as independent variables.

**Results:**

Positive correlations have been identified between internalized and relational ageism (r = 0.41, *p* < 0.01) and internalized and relational ableism (r = 0.50, *p* < 0.01), suggesting that internalized and relational variants of each bias coexist. The results showed strong cross-construct correlations between ageism and ableism subscales (r = 0.26–0.40, *p* < 0.01). Among four regression models, ageism and ableism subscales were significantly associated with each other (R^2^ = 0.140–0.226, all *p* < 0.001), Affinity for Older People was significantly associated with relational ageism (*β* = 0.226, *p* = 0.001). Younger age was the only demographic variable significantly associated with outcomes across models, linked with higher levels of internalized ableism (*β* = −0.156, *p* = 0.038). Gender, year of study, and prior experience were not significant in any of the models.

**Conclusion:**

Among undergraduate medical students, ageism and ableism are closely related, with attitudinal indicators outperforming demographic ones. In order to address both biases concurrently in undergraduate medical education and advance fair, inclusive patient care, these findings underscore the critical need for focused educational interventions, such as structured experiential learning, reflective practice, and disability-focused curriculum content.

## Introduction

Ageism, first defined as prejudice by one age group against other age groups (1), and then expanded into a broad system of negative and positive stereotypes directed at older adults (2), is a common bias that has a substantial impact on healthcare delivery. Similarly, ableism, which is described as a network of beliefs and behaviors that generates a specific type of body and self as the ideal standard (3), and operates across numerous levels of society in addition to disability (4), is an equally prevalent form of discrimination. Ageism and ableism work together to influence attitudes, actions, and equitable healthcare delivery (5). These biases can interfere with the quality of care provided for vulnerable populations in clinical settings, particularly older adults and people with disabilities (6). These biases may lead to under-treatment, exclusion from clinical decision-making, and generally worse patient experience (7). Age discrimination in healthcare has been found to increase the risk of depression, lower compliance, and poor health outcomes. Ageism and ableism are two interrelated forms of discrimination (8). Ageism and ableism may be linked by a shared underlying logic: both devalue individuals who deviate from an idealized standard of youth, productivity, and physical capacity. Stereotypes equating older age with declining ability and dependence may serve as a common cognitive basis linking age-based and disability-based prejudice, such that individuals who endorse one form of bias are predisposed to endorse the other.

Ageism includes age-based stereotypes and prejudices that are typically aimed at older adults, whereas ableism relates to negative views regarding disability and the notion that disability is undesirable or unnatural. In the medical field, these biases commonly coexist, particularly for older adults with disabilities who may be doubly marginalized (9). For instance, an older adult with mobility issues may be perceived as weak and unable, which may have an impact on the type and quality of care they receive (10). Understanding how the two forms of discrimination interact is necessary to promote empathy and reduce biases in clinical practice (11). As future healthcare providers, healthcare students’ perceptions of ageism and ableism might influence their future practice (12). Ageist views, such as overlooking older patients’ complaints, removing them from decision-making, or deprioritizing their treatment needs, have been shown to result in inferior care (13). Furthermore, considering the growing need and shortage of geriatric care professionals, there are still a few medical professionals with training in gerontology and geriatrics (14). In the United States, only three of the nation’s 145 medical schools include geriatrics departments, and less than 10% of them require a geriatrics course, highlighting a serious shortage of geriatrics-prepared health care providers (15).

Due to the lack of doctors with training in geriatric medicine, it has been demonstrated that other medical specialists, such as geriatric advanced practice nurses, can learn many of the specialized skills that geriatricians typically offer, providing a long-term solution to the care needs of frail older adults.

Several studies have examined ageism among healthcare and pre-healthcare workers (16, 17). Ageism may contribute to the shortage of these professions. If these prejudices are not addressed in education, the future healthcare professionals might lack the knowledge and empathy necessary to offer good care to older adults (18). Reducing these biases has been demonstrated to be possible with interventions including interactive seminars, focused training, and satisfying clinical experiences with older adults (19). Educational institutions can better train students to provide inclusive, respectful, and equitable treatment to all patients, regardless of age or ability, by integrating content related to ageism and ableism into healthcare education (20). However, research on healthcare students’ perceptions of ageism and ableism remains limited.

In this study, we distinguish between the internalized and relational aspects of each bias. Internalized ageism and internalized ableism refer to the self-directed acceptance of negative age- or disability-related stereotypes for example, predicting that one’s own worth, independence, or capability will deteriorate with age or disability. Relational ageism and relational ableism, by contrast, refer to fear about being adversely appraised, disregarded, or undervalued by others on the basis of age or disability, rather than prejudice the individual expresses against others (21). This distinction allows us to study both self-directed and interpersonally-anticipated features of ageism and ableism as conceptually different but linked phenomena”.

In this study, we aimed to examine the Factors associated with ageism and ableism among undergraduate medical students in China and to explore the interrelationships between internalized and relational forms of these biases. Specifically, we addressed the following research questions:

RQ1: What are the levels of ageism and ableism among undergraduate medical students at a Chinese university?

RQ2: Which demographic factors (age, gender, year of study, and prior experience with older adults or people with disabilities) are associated with internalized and relational ageism among undergraduate medical students?

RQ3: Which demographic factors are associated with internalized and relational ableism among undergraduate medical students?

RQ4: To what extent are ageism-related attitudes associated with ableism outcomes, and vice versa, after controlling for demographic factors?

## Methods

### Study design

We adopted a cross-sectional design and collected survey data in November 2024.

### Settings and participants

This study was conducted at Qingdao University, a comprehensive public university in the city of Qingdao, Shandong province, in northeastern China. The university was selected because of its large enrolment of medical undergraduate students, providing an appropriate population for investigating perceptions of ageism and ableism among future healthcare professionals. Participants were selected from the medical undergraduate program, and convenience sampling was used.

Eligible participants were international students from Asian countries currently enrolled in the English-medium undergraduate medical program at Qingdao University, China. Students from non-medical majors were excluded. Emails and announcements in the classroom were used to recruit participants from approximately 500 eligible students. 199 of the approximately 500 eligible students who were invited finished the survey, resulting in a response rate of about 40%. Convenience sampling was used for recruitment, and participation was anonymous and voluntary.

Undergraduate medical education in China is a five-year program that begins immediately after secondary school, in contrast to North American medical systems, where medical school follows a bachelor’s degree. Graduates can become licensed clinicians by taking the National Medical Licensing Examination. Because of this, the college years are crucial for forming professional attitudes, including those about ableism and ageism.

### Instruments

Table 1 summarizes measures used in this study. We developed questions to assess demographic and background information, including age, gender, year of study, prior experience interacting with older adults or those with disabilities, and exposure to gerontology or education related to disability (22). All five subscales Affinity for Older People, Internalized Ageism, Relational Ageism, Internalized Ableism, and Relational Ableism were generated from the Relational Aging Anxiety Scale, which was created and validated among adults in the United States (23). This instrument was constructed around the conceptual junction of ageism and ableism, so it includes ageism- and ableism-focused subscales that measure both the internalized (self-directed) and relational (other-directed/anticipated) elements of each prejudice. The ableism subscales (Internalized Ableism: 6 items) Relational Ableism (four items) mirrors the structure of the ageism subscales, assessing identical self-directed and relationally expected concerns in the context of disability rather than age.

**Table 1:** Summary of measures used in the study.

Construct	Definition		Items	Sample item	Response options	Scoring	Cronbach’s *α*
Ageism and ableism							
Affinity for older people	Favorable attitudes, enjoyment, and comfort in interacting with older adults.	5 items from the Relational Aging Anxiety Scale	I enjoy being around older people.	5-point Likert Scale from strongly agree to strongly disagree		Mean score (1-5): Sum of 5 items divided by 5	0.763
Internalized ageism	Acceptance of negative beliefs about aging applied to oneself.	5 items from the Relational Aging Anxiety Scale	I worry that there will be no meaning in life in my older age.	5-point Likert Scale from strongly agree to strongly disagree		Mean score (1-5) Sum of 5 items (reverse-scored where needed) divided by 5	0.429
Relational ageism	Concerns about being judged or devalued by others due to age.	6 items from the Relational Aging Anxiety Scale	My opinions will not matter to others in my older age.	5-point Likert Scale from strongly agree to strongly disagree		Mean score (1-5) Sum of 6 items (reverse-scored where needed) divided by 6	0.637
Relational ableism	Expected devaluation or stigmatization by others as a result of impairment status.	4 items from the Relational Ableism Scale	If I use a wheelchair, others will see me as less valuable.	5-point Likert Scale from strongly agree to strongly disagree	Mean score (1-5): Sum of 4 items divided by 4		0.833
Internalized Ableism	Internal fears and self-stigma related to potential disabilities.	6 items from the Internalized Ableism Scale	I worry that if I have a disability, I will be a burden to others.	5-point Likert Scale from strongly agree to strongly disagree		Mean score (1-5): Sum of 6 items divided by 6	0.861
Demographics							
Age	The age of the participant in years.		1	What is your age?	Open ended		N/A
Gender	Participant’s self-identified gender.		1	What is your gender?	Male/female/ non-binary/prefer not to say		N/A
Grade Level	Participant’s academic year in the medical program.		1	What is your grade level?	1st year/2nd year/3rd year/4th year/5th year		N/A
Major	Medicine		1	What is your major in health care professions?	Medicine/public health/pharmacy/nursing/other (specify)		N/A
Prior Experience with older adults	Previous interactions with older adults		1	Have you had prior experience with older adults?	None/volunteer work/paid work/family caregiving/other (specify)		N/A

The Relational Aging Anxiety Scale was used as the base for all subscales (23). The current sample’s internal consistency appears in the Cronbach’s α values.

The Relational Aging Anxiety Scale was administered in its original English-language form, a decision based on the specific linguistic context of the study setting. English is the primary medium of instruction for the undergraduate medical curriculum at Qingdao University; students are required to read core medical textbooks and primary literature, attend English-medium lectures, and complete written examinations in English from their first year onward. All participants therefore possessed advanced academic English proficiency sufficient to comprehend the conceptual content of the scale items, which employ everyday rather than technical vocabulary. To verify that the instrument functioned reliably in this population, we examined its internal consistency in the present sample. All Subscale reliability varied (*α* = 0.429–0.861). While Internalized Ageism (α = 0.429) and Relational Ageism (α = 0.637) fell below the standard. 70 criterion, three subscales (Affinity for Older People, Internalized Ableism, and Relational Ableism) showed acceptable-to-good internal consistency (α ≥ 0.70). Results involving these two subscales be interpreted cautiously throughout because they are used as both predictors and outcomes in the regression models.

Comparable to values reported in the original validation study, indicating that the items cohered as intended among Chinese medical students. We acknowledge, however, that adequate language proficiency does not by itself guarantee full conceptual and cross-cultural equivalence; this issue is addressed further in the Limitations section.

Five subscales, affinity for older people, internalized ageism, relational ageism, internalized ableism, and relational ableism, assessed multiple aspects of ageism and ableism. A 5-point Likert scale was used to record the responses (1 being strongly agree, and 5 being strongly disagree). A mean score (1–5) was calculated by adding up and averaging the item responses (24). Below, we introduce each of the five subscales.

### Affinity for older people

The construct Affinity for Older People describes the favorable attitudes, feelings, and actions people have toward older adults. It includes a person’s readiness, comfort level, and enjoyment of interacting with older people in a variety of settings (23). As a result the items on the Affinity for Older People subscale were positively worded (e.g., “I enjoy being around older people”) and the response scale ranged from 1 (strongly agree) to 5 (strongly disagree), higher scores on this subscale indicate lower affinity for older people (i.e., greater disagreement with favorable statements), not higher affinity, unlike the ageism and ableism subscales.

A sample item from this subscale is “I enjoy spending time with older people.” The subscale demonstrated Acceptable internal consistency in the current sample (*α* = 0.763).

### Internalized ageism

Accepting unfavorable social preconceptions about aging that people either apply to themselves or anticipate as they get older is one example of aging. It can show itself as concern over losing one’s ability, independence, or value, fear of aging, or poor self-perceptions. Internalized ageism may affect how healthcare students view older patients, which could lower their empathy and interest in geriatric care.

Items that gauge attitudes, expectations, and fears related to aging, such as worries about life happiness or one’s own worth as one ages, are commonly used to evaluate this construct. Its impacts can be lessened, and more equal attitudes in healthcare can be promoted by addressing internalized ageism through education and constructive encounters with older persons (25).

This subscale’s sample item is “I worry that there will be no meaning in life in my older age.” The subscale demonstrated poor internal consistency in the current sample (*α* = 0.429).

### Relational ageism

Relational ageism, as compared to self-directed internalized ageism, is the evaluation of individuals by others according to their age, with an emphasis on perceived attitudes and exterior interactions. It includes worries of being ignored, mistreated, or considered to be less competent because of one’s age. The customized questionnaire, derived from validated measures, examined this construct using five items, such as judgments of respect and valuing of older adults’ contributions (26).

This subscale includes items such as “My opinions will not matter to others in my older age.” The subscale demonstrated questionable internal consistency in the current sample (*α* = 0.637).

### Internalized ableism

Internalized ableism is utilized to describe how people who have or expect to have a disability accept the prejudices and stereotypes about disabilities that society has about them. Because of physical, sensory, or cognitive deficits, this phenomenon can show up as dread, guilt, or self-doubt about one’s abilities, worth, or quality of life (27). People who have internalized ableism may believe they are a burden, incompetent, or unfit to participate in worthwhile activities or connections.

Internalized ableism may affect how healthcare students view disability and how they feel about patients with disabilities, which could result in biased treatment (28). Items examining worries about being a burden, a lower quality of life, and fears of stigma are commonly used to measure this concept. Promoting inclusive attitudes in healthcare practice, raising awareness of ableist biases, and encouraging positive narratives surrounding disability are all important components of combating internalized ableism.

This subscale’s example item is “I worry that if I have a disability, I will be a burden to others.” The subscale demonstrated good internal consistency in the current sample (*α* = 0.861).

### Relational ableism

People who believe or expect that they will be treated differently, judged, or devalued by others due to their disability status are an aspect of this concept. It expresses worries about being stigmatized, ostracized, or viewed as less competent, useful, or worthy of respect because of cognitive, sensory, or physical disabilities. Relational ableism emphasizes the expected or experienced attitudes of others in social and professional relationships, as opposed to internalized ableism, which concentrates on self-directed biases (29).

Relational ableism can influence how healthcare students approach patient care by forming their perception of how society perceives and handles people with impairments. Items assessing perceived exclusion, lack of acknowledgment, or devaluation based on disability are commonly used to evaluate it. Fostering inclusive attitudes, dispelling social preconceptions, and advocating for respect and equity for people with disabilities in healthcare and other fields are all part of addressing relational ableism.

This subscale’s sample item is “If I use a wheelchair, others will see me as less valuable.” The subscale demonstrated good internal consistency in the current sample (*α* = 0.833).

### Data collection procedure

The research project received ethical approval from Qingdao University’s Medical College ethics committee (Protocol code QDU-HEC-2024339). Over a period of three weeks, announcements in the classroom and emails were used to attract undergraduate medical students. Before their involvement in the study, each participant reviewed an online consent form and provided informed consent to participate in the study.

To provide easy accessibility and participation for all students, an online questionnaire delivered through Google Forms was used for data collection. Every participant filled out the survey completely, and so we did not have any missing values for any of the variables. The online questionnaire’s mandatory response boxes helped to ensure the completeness of the data.

## Statistical analysis

Given that the primary objective of this study was to identify factors associated with ageism and ableism, these constructs were treated as dependent variables in separate regression models. All statistical analyses were performed using Python (version 3.x) with the scikit-learn, pandas, scipy, and statsmodels packages. The significance level was set at *p* < 0.05. Analyses were conducted sequentially as follows:

### Descriptive analyses of demographics

Descriptive statistics were used to characterize the participants’ age, gender, academic year, and previous experience working with older adults or individuals with disabilities.

### Correlation analysis of subscales

Pearson’s correlation coefficients were used to analyze relationships between pairs of the five Relational Aging Anxiety subscales (Affinity for Older People, Internalized Ageism, Relational Ageism, Internalized Ableism, and Relational Ableism). Before proceeding to multi-variable modeling, this analysis informed the strength of the correlations between the main constructs.

### Internal consistency of subscales

Cronbach’s alpha was used to assess internal consistency; *α* > 0.70 indicated acceptable reliability, α ≥ 0.80 indicated strong reliability, and α ≥ 0.90 denoted exceptional dependability.

### Regression analysis

The factors of ageism and ableism among healthcare students were investigated using four sets of multiple regression models. Internalized Ageism and Relational Ageism were the dependent variables in two different regression models. Age, gender, year of study, previous experience interacting with older persons or people with impairments, and the two ableism subscales (Relational Ableism and Internalized Ableism) were the independent variables.

Internalized Ableism and Relational Ableism were the dependent variables in two other regression models. The three ageism subscales (Affinity for Older People, Internalized Ageism, and Relational Ageism), as well as age, gender, year of study, and previous experience engaging with older adults or people with disabilities, were the independent variables. We realize that the predictors and outcomes in these models are subscales of the same parent instrument. This modeling technique was chosen purposefully and is consistent with previous research on the interrelationships between ageism and ableism constructs, in which the fundamental question is how these attitudinal domains connect to one another rather than predicting an external criterion. Because our goal was to quantify the strength and direction of associations between internalized and relational forms of each bias while controlling for demographic characteristics rather than to build a predictive model of an independent outcome, we used conceptually related subscales as both predictors and outcomes, reflecting the study’s exploratory objectives. To reduce the possibility that the observed associations were artifacts of collinearity, we examined the bivariate correlations among all subscales before modeling (Table 2); inter-subscale correlations were low to moderate (r = 0.07–0.50), indicating that the constructs are related but empirically distinct.

**Table 2:** Pearson correlation matrix of ageism and ableism subscales (*N* = 199).

Subscale	1	2	3	4	5	M	SD
1. Affinity for older people	—					10.04	2.72
2. Internalized ageism	0.17*	—				12.58	2.64
3. Relational ageism	0.26**	0.41**	—			14.76	2.91
4. Relational ableism	0.07	0.33**	0.40**	—		11.45	3.51
5. Internalized ableism	0.07	0.30**	0.26**	0.50**	—	15.22	4.58

M = Mean. SD = Standard Deviation. * *p* < 0.05. ** *p* < 0.01. All subscales derived from the Relational Aging Anxiety Scale (23).

To evaluate multicollinearity, Variance Inflation Factors (VIF) were calculated for each predictor in all four regression models.

Because all independent and dependent variables were collected from the same participants via a single self-report instrument, Harman’s single-factor test was used to check common method bias. All 26 items were included in an unrotated principal component analysis. The first component accounted for 21.97% of the total variance, which is significantly below the 50% criterion, demonstrating that common technique bias was not a major concern in the current study. The Limitations section explores how this strategy affects shared method variance and conceptual overlap.

### Sample size

The number of eligible and consenting students available during the recruitment window determined the sample size; no *a priori* power analysis was done before data collection. We utilized Green’s popular multiple regression guidelines, which suggest a minimum of N ≥ 50 + 8 m for testing the entire model and N ≥ 104 + m for testing individual predictors, where m is the number of predictors, to assess the sufficiency of the obtained sample *post hoc*. These criteria demand minimum samples of roughly 106 and 111, respectively, with a maximum of seven predictors per model.

To assess sample adequacy, a post-hoc power analysis was performed for each regression model (*α* = 0.05, *N* = 199), taking into account the number of predictors (6, 7) and residual degrees of freedom. This showed 80% power to identify effect sizes as small as f^2^ = 0.071–0.075, which corresponds to a small-to-medium effect based on established benchmarks (44). The current study’s effect sizes (f^2^ = 0.163–0.292, estimated from model R^2^) exceeded the threshold in all four models, showing that the sample size was sufficient to detect the reported connections.

## Results

### Descriptive results

Table 3 provides a detailed summary of the demographic characteristics and descriptive statistics. A total of 199 medical undergraduate students participated in the study. Males made up the majority of the 199 survey respondents (157, 78.9%), followed by females (38, 19.1%). Just a small number of people chose not to reveal their gender (3, 1.5%) or identified as non-binary/third gender (1, 0.5%). The group of respondents appeared to be extremely youthful since a majority of participants (141, 70.9%) were between the ages of 20 and 24. Participants came from a variety of academic years, but first-year students made up the largest percentage (52, 26.1%), followed by third-year students (47, 23.6%) and fourth-year students (37, 18.6%). There were no participants from other healthcare disciplines; all were enrolled in medical programs.

**Table 3:** Demographic characteristics of participants.

Demographic characteristics of participants	*N* (%)
Gender	
Male identified	157 (78.9)
Female identified	38 (19.1)
Non-binary/Third identified	1 (0.5)
Prefer not to say identified	3 (1.5)
Age	
Under 20	33 (16.6)
20–24	141 (70.9)
25–29	22 (11.1)
30 and above	3 (1.5)
Grade level	
First-year	52 (26.1)
Second year	36 (18.1)
Third year	47 (23.6)
Fourth year	37 (18.6)
Fifth year	27 (13.6)
Major	
Medicine	199 (100.0)
Previous experience with older adults or individuals with disabilities	
None	82 (41.2)
Volunteer work	45 (22.6)
Paid work	6 (3.0)
Family care	60 (30.2)
Other*	6 (3.0)

*N* = 199. *Other includes interactions in community or clinical settings.

Although a significant majority of participants (82, 41.2%) reported having no prior experience interacting with older adults or people with disabilities, the most common forms of exposure were family caregiving (60, 30.2%) and volunteer work (45, 22.6%). Participants generally expressed positive opinions about older people, saying that they felt at ease and enjoyed their company. Among the issues raised by the participants were concerns about aging, being ignored, or experiencing prejudice because of a disability or the use of mobility aids.

### Internal consistency

Cronbach’s alpha was used to evaluate each subscale’s internal consistency. All five Subscales showed variable reliability; not all exceeded α > 0.70 (Internalized Ageism α = 0.429; Relational Ageism α = 0.637), with two subscales falling below acceptable thresholds (Table 1).

#### Correlation analysis among subscales of ageism and ableism

The Pearson correlation matrix for each of the five subscales is shown in Table 2. Relational and internalized variations of each prejudice tend to coexist, as evidenced by significant positive correlations between internalized ageism and relational ageism (r = 0.41, *p* < 0.01) and between internalized ableism and relational ableism (r = 0.50, *p* < 0.01). While its connections with both ableism subscales were minimal and non-significant (r = 0.07, *p* > 0.05),

Relational ageism (r = 0.26, *p* < 0.01) and internalized ageism (r = 0.17, *p* < 0.05) showed a positive correlation with affinity for older people. These positive correlations show that lower affinity for older people is linked to more internalized and relational ageism because higher Affinity scores reflect fewer positive attitudes toward older people (see Instruments). This pattern is consistent with theoretical expectations linking negative attitudes toward older adults to greater personal age-related anxiety.

### Regression analysis

To find factors that influence ageism and ableism outcomes, four multiple regression models were constructed. Table 4 displays the results for every model.

**Table 4:** Summary of multiple linear regression results for ageism and ableism models (*N* = 199).

Predictor variable	Model A1 internalized ageism β	Model A2 relational ageism β	Model B1 internalized ableism β	Model B2 relational ableism β
Demographic predictors				
Age	−0.093	−0.046	−0.156*	−0.071
Gender	−0.038	0.087	−0.035	0.041
Year of study	0.053	0.040	0.136	0.079
Prior experience	0.089	−0.011	0.003	−0.101
Attitudinal predictors				
Affinity for older people	—	0.226**	−0.018	−0.042
Internalized ageism	—	—	0.218**	0.209**
Relational ageism	—	—	0.152*	0.305***
Relational ableism	0.251**	0.336***	—	—
Internalized ableism	0.157*	0.060	—	—
Model statistics				
R^2^	0.151	0.226	0.140	0.215
Adjusted R^2^	0.124	0.197	0.108	0.186
F-statistic	5.685***	7.961***	4.430***	7.461***
df	6, 192	7, 191	7, 191	7, 191
Max VIF	1.39	1.39	1.31	1.31
Durbin-Watson	1.74	1.93	1.76	1.91
*p*	<0.001	<0.001	<0.001	<0.001

*N* = 199. β = standardized regression coefficient. Model A1: DV = Internalized Ageism. Model A2: DV = Relational Ageism. Model B1: DV = Internalized Ableism. Model B2: DV = Relational Ableism. — = predictor not included in model. * *p* < 0.05; ** *p* < 0.01; *** *p* < 0.001. Max VIF = maximum Variance Inflation Factor among predictors in each model; all VIF values ranged from 1.03 to 1.39 across all models, well below the threshold of 5 (45), confirming no multicollinearity. Durbin-Watson values between 1.5 and 2.5 indicate no problematic autocorrelation. Shapiro–Wilk *p* > 0.05 indicates residual normality (Model B1 *p* = 0.008 suggests slight non-normality, but OLS is robust with *N* = 199). Cook’s distance < 4/n confirms no influential outliers. Homoscedasticity confirmed via residual-versus-fitted plots. Bold values in this table indicate statistically significant predictors (*p* < 0.05).

Factors associated with internalized ageism (Model A1). The total model explained 15.1% of the variance in internalized ageism and was statistically significant (R^2^ = 0.151, Adjusted R^2^ = 0.124, *F* (6, 192) = 5.685, *p* < 0.001). Age (*β* = −0.093, *p* = 0.213), gender (*β* = −0.038, *p* = 0.575), year of study (*β* = 0.053, *p* = 0.463), and past experience (*β* = 0.089, *p* = 0.185) were not significant demographic predictors. Relational Ableism (*β* = 0.251, *p* = 0.002) and Internalized Ableism (*β* = 0.157, *p* = 0.046) were significantly and positively associated with internalized ageism among attitudinal variables, suggesting that higher levels of ableism were linked to more internalized ageism.

Factors associated with relational ageism (Model A2). The total model explained 22.6% of the variance in relational ageism and was statistically significant (R^2^ = 0.226, Adjusted R^2^ = 0.197, *F* (7, 191) = 7.961, *p* < 0.001). Age (*β* = −0.046, *p* = 0.518), gender (*β* = 0.087, *p* = 0.186), year of study (*β* = 0.040, *p* = 0.567), and past experience (*β* = −0.011, *p* = 0.867) were not significantly associated with the outcome.

Affinity for Older People (*β* = 0.226, *p* = 0.001) and Relational Ableism (*β* = 0.336, *p* < 0.001) were both significantly associated with relational ageism; according to the Affinity subscale’s score direction, lower affinity for older people was linked to higher relational ageism.

Internalized Ableism (*β* = 0.060, *p* = 0.422) was not a significantly associated with the outcome.

Factors associated with internalized ableism, Model B1. The total model explained 14.0% of the variance in internalized ableism and was statistically significant (R^2^ = 0.140, Adjusted R^2^ = 0.108, *F* (7,191) = 4.430, *p* < 0.001). The only demographic variable significantly associated with the outcome was age (*β* = −0.156, *p* = 0.038), suggesting that students who were younger had higher levels of internalized ableism. Prior experience (*β* = 0.003, *p* = 0.965), year of study (*β* = 0.136, *p* = 0.062), and gender (*β* = −0.035, *p* = 0.612) were not significant. Internalized ableism was significantly positively predicted by Relational Ageism (*β* = 0.152, *p* = 0.048) and Internalized Ageism (*β* = 0.218, *p* = 0.004) among attitudinal Variables. There was no significant affinity for older people (*β* = −0.018, *p* = 0.803).

Factors associated with relational ableism, Model B2. The total model explained 21.5% of the variance in relational ableism and was statistically significant (R^2^ = 0.215, Adjusted R^2^ = 0.186, *F* (7, 191) = 7.461, *p* < 0.001). Age (*β* = −0.071, *p* = 0.321), gender (*β* = 0.041, *p* = 0.540), year of study (*β* = 0.079, *p* = 0.257), and past experience (*β* = −0.101, *p* = 0.119) were not significant predictors. Relational ableism was significantly positively associated with internalized ageism (*β* = 0.209, *p* = 0.004) and relational ageism (*β* = 0.305, *p* < 0.001). There was no significant affinity for older people (*β* = −0.042, *p* = 0.534). Ageism and ableism subscales consistently and significantly predicted one another across all four models, demonstrating how closely related they are among undergraduate medical students. Multicollinearity diagnostics indicated that VIF values ranged from 1.03 to 1.39 across all four models, significantly below the traditional threshold of 5, indicating that multicollinearity was not an issue.

### Reduced exploratory models

Reduced models that only included the relevant attitudinal factors were fitted for each outcome in order to determine whether the substantial attitudinal correlations remained stable after excluding non-significant demographic covariates. For Internalized Ageism, Relational Ableism (*β* = 0.238, *p* = 0.002) and Internalized Ableism (*β* = 0.173, *p* = 0.026) continued to be significant. Relational Ableism (*β* = 0.348, *p* < 0.001) and Affinity for Older People (*β* = 0.219, *p* = 0.001) continued to be significant for Relational Ageism. In a similar vein, the attitudinal factors that were significant in the primary models for Models B1 and B2 continued to be significant in the reduced models, with magnitude and direction in line with the primary analysis shown in Table 4. Although they do not represent a formal robustness or sensitivity test, these simplified models imply that the attitudinal correlations were independent of the particular set of demographic covariates included.

## Discussion

Significant positive correlations between internalized and relational versions of each bias were found (r = 0.41 for ageism, r = 0.50 for ableism, *p* < 0.01), confirming the close relationship between ageism and ableism among undergraduate medical students (30). These correlations imply that students’ prejudices are not isolated viewpoints but rather represent larger cultural narratives about aging and disability that are psychologically acquired throughout training. This interpretation aligns with the Stereotype Embodiment Theory (31), which maintains that stereotypes about age and disability that are common in society are internalized throughout life and function as self-relevant beliefs that influence expectations, feelings, and behavior. Our discovery that internalized and relational forms of each bias co-occur suggests that these broader cultural narratives are acquired early and may become embedded in emerging professional identity. In a demanding medical curriculum, students absorb not only clinical knowledge but also implicit norms and hierarchies of the profession.

Ageism and ableism subscales were consistently and significantly associated with each other across all four regression models (R^2^ = 0.140–0.226), highlighting the need for educational initiatives that address both biases concurrently rather than separately. It should be noted, however, that these models accounted for only a small proportion of the variance in each outcome (14.0–22.6%), implying that most of the variance in ageist and ableist attitudes is likely due to factors not captured here, such as curriculum content, clinical exposure, cultural narratives, personality dispositions, and implicit bias. The relationships shown below should thus be interpreted as identifying significant but partial factors to these attitudes rather than entire explanatory models.

Affinity for Older People was substantially correlated with relational ageism (*β* = 0.226, *p* = 0.001). This finding suggests that students who reported less positive attitudes toward older adults also reported greater relational ageism concern, which is consistent with expectations that more positive intergroup attitudes are associated with lower age-based bias because higher scores on this subscale reflect lower affinity (see Instruments). This result is consistent with the intergroup contact theory (32), which postulates that good interactions and comfort with older adults may operate as a buffer against internalized and relationally predicted age-based devaluation.

Which proposes that persistent, positive contact with older persons may increase students’ sensitivity to, and recognition of, the age-based devaluation that older people face in social and institutional settings. In this interpretation, affinity does not cause relational ageism but rather raises awareness of it. Students who feel close to older individuals may be more likely to identify and report relational slights and status threats associated with aging. A non-exclusive possibility is that the relational ageism subscale includes anticipatory concern about one’s own future ageing, which may be more prevalent among people who interact closely with older adults. Both interpretations suggest that affinity and awareness of ageism are not mutually exclusive, and that contact-based therapies should be combined with explicit, organized reflection to translate increased awareness into reduced bias.”

To lessen ageist views and increase empathy for older patients, this study emphasizes the significance of encouraging constructive, organized intergenerational contact in medical education (33). The only significant demographic predictor in all models was younger age, which was linked to higher levels of internalized ableism (*β* = −0.156, *p* = 0.038). This suggests that younger students may be more prone to internalizing stigma related to disabilities, perhaps because of having less exposure to structured disability education or diverse life experiences (34). From a developmental perspective, younger students might be at an earlier stage of developing the professional schemas through which disability is subsequently understood, and they might have had fewer opportunities for the kind of meaningful interaction with individuals with disabilities that tends to lessen internalized stigma.

This pattern suggests that anti-ableism content may be particularly crucial in the early years of training, before stigmatizing schemas solidify, and it is consistent with data that disability attitudes become more nuanced with age, clinical exposure, and focused education.

This finding highlights the complexity of age-related attitudinal patterns and the necessity for focused treatments across all year levels in medical training, in contrast to some research that suggests younger people have stronger ageist attitudes (35). In contrast to the gender-balanced or female-majority composition typical of North American medical programs, the study sample was predominantly male (78.9%) (36). This is typical of Asian undergraduate medical curricula and represents the actual enrolment pattern at Qingdao University. Crucially, gender did not significantly predict ableism or ageism (*p* = 0.575) in any model, indicating that these attitudes may not be highly dependent on gender. However, the findings may not be as broadly applicable due to the low representation of female students and the greater percentage of first-year students (26.1%) with little clinical experience (37).

To further understand how views change during medical training, future research should use stratified or gender-balanced samples from various year levels (38). No regression model found that prior experience working with older persons or people with disabilities was a significant predictor (*p* > 0.05). This contradicts presumptions that exposure to these groups alone can lessen prejudice and is in line with data indicating that interaction quality, not quantity, is the primary factor influencing changes in attitudes toward older adults and those with disabilities (39).

**Figure 1:**
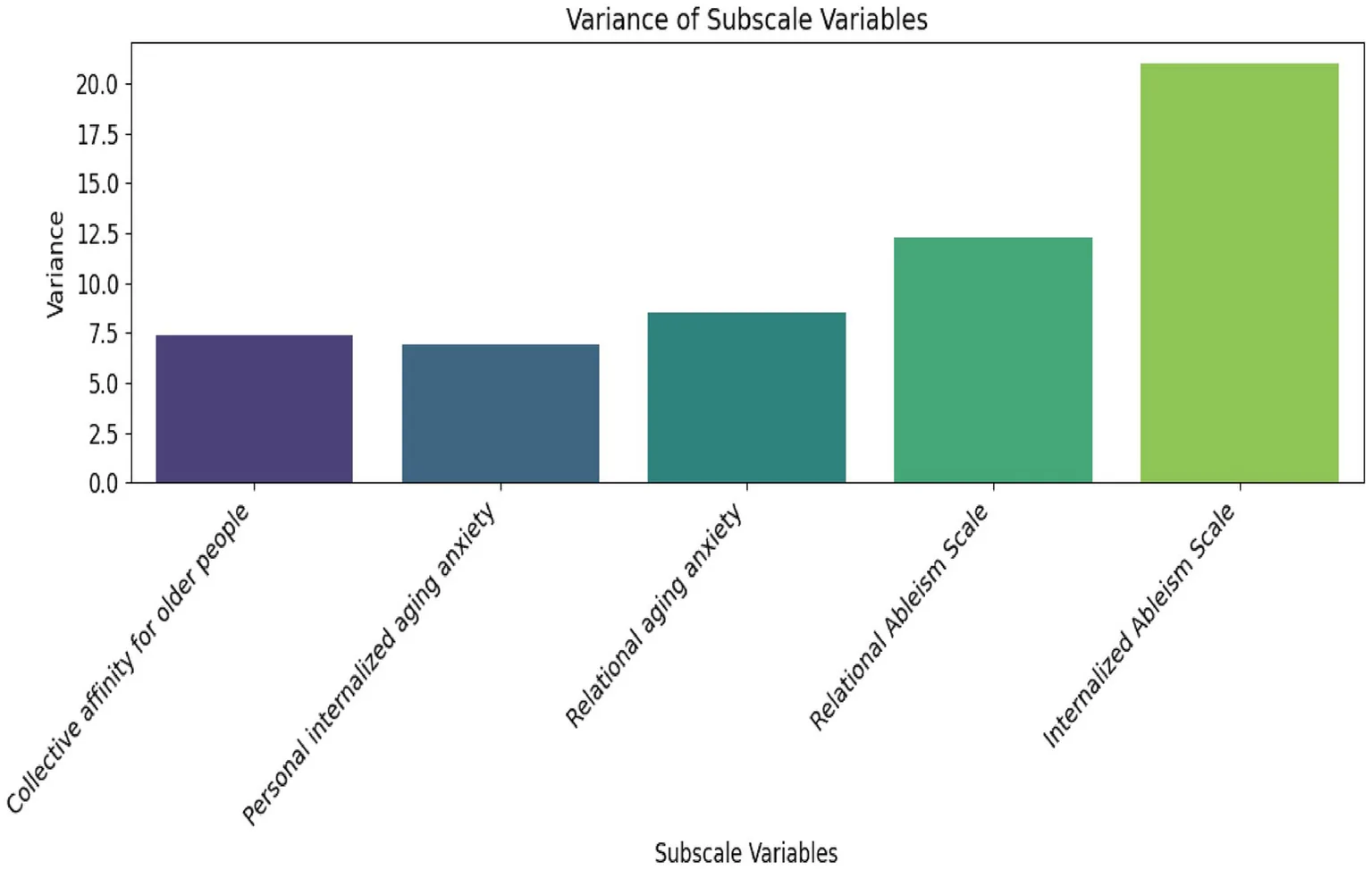
Variance in subscale scores for ageism and ableism measures among 199 undergraduate medical students. Variance values are calculated using total summed subscale scores.

Therefore, rather than only increasing the frequency of contact, future educational interventions should concentrate on the quality of student-patient interactions, such as supervised clinical encounters and structured reflective practice (40). Students’ perspectives on disability appear to be more diverse than their perspectives on aging, as seen by the higher variety in attitudes toward disability compared to ageism (Figure 1). The complex and multifaceted nature of disability stigma, disparate individual experiences with impairment, or unequal representation of disability content in the curriculum could all be contributing factors (41). This heterogeneity suggests that ageism-focused measurements alone may not be sufficient for ableism therapies; instead, more customized and nuanced methods may be needed.

### Implications for healthcare education and workforce development

These findings have major implications for healthcare education and workforce development. First, early and targeted interventions, beginning in the first year of medical school, may help students maintain good attitudes throughout their studies. Second, systematic experiential learning, such as standardized geriatric patient encounters and disability simulation exercises, can address both relational and internalized types of bias and hence should be more widely included in the medical curriculum. Third, because ageism and ableism are inextricably linked, curriculum designers should employ a dual-focus technique that addresses both biases simultaneously. Future research should evaluate the effectiveness of combined ageism and ableism interventions in creating long-term attitudinal change, as well as use longitudinal designs to examine how attitudes change over the course of medical education.

Disability stigma is complicated and multidimensional, and there is unequal representation of disability information in medical curricula, which may account for the wider range of views toward disability compared to ageism (42).

Our findings are consistent with an increasing amount of research that views ableism and ageism as overlapping, mutually reinforcing systems of bias rather than separate attitudes (4, 36, 37). Both are based on a similar logic that devalues people who deviate from an idealized “young” and “able” norm by equating human worth with youth, productivity, and physical capacity. This shared basis explains why older persons with impairments, who sit at the crossroads of both, are more likely to experience compounded marginalization in healthcare, and why students who supported one type of bias also tended to support the other. The claim that the two biases are best addressed in tandem is supported by interpreting our results via this intersectional lens (43).

Prior experience’s non-significant impact on all four models (all *p* > 0.05) indicates that interaction quality rather than exposure amount alone is more crucial in determining students’ attitudes.

### Limitations

This study has several limitations. First, a longitudinal study is required to monitor changes in attitudes during medical training, as the cross-sectional design prevents causal inference. Second, the use of convenience sampling at a single university, a sample that is primarily male (78.9%), a higher percentage of first-year students (26.1%), and a response rate of almost 40% all limit generalizability. Gender was assessed as a covariate in all regression models and was not a significant predictor in any model, indicating that the gender imbalance did not significantly affect the results. To allay these worries, demographic distributions were provided openly. Future study should incorporate multi-site designs with a stratified sample to establish generalizability. Third, social desirability bias could be introduced via self-report measures. Fourth, R^2^ values (0.140–0.226) show that the majority of variance is still not explained by the variables measured, most likely because of unmeasured elements like implicit biases, cultural narratives, and curriculum content. As a result, the results should be interpreted as identifying important but incomplete contributors to these attitudes.

Fifth, the same parent instrument’s subscales were employed as outcomes and predictors. These subscales, however, measure ageism and ableism, two conceptually separate categories that have been theoretically and empirically distinguished in the literature. To address potential methodological issues, variance inflation factor (VIF) values confirmed that multicollinearity was not a concern in any of the four models (1.03–1.39), and Harman’s single-factor test indicated that common method bias was not a significant concern (first factor = 21.97%, significantly below the 50% criterion).

However, it is important to consider observed impact sizes as upper-bound estimates that may partially account for shared method variance. The Sixth is the original cross-cultural adaptation. Language competency does not ensure conceptual equivalency, even though all participants were international students with outstanding academic English proficiency. Two subscales (Internalized Ageism *α* = 0.429; Relational Ageism α = 0.637) had internal consistency below the 0.70 criterion, which may indicate mixed item directionality and cultural diversity in the interpretation of aging-related questions. Reliability values were provided for all subscales in order to account for this, and results pertaining to these two subscales were interpreted cautiously. Before using this tool with populations from around the world, future studies should conduct rigorous cross-cultural adaptation and measurement-invariance testing.

## Conclusion

Internalized and relational forms of ageism and ableism are closely related and consistently associated with one another across all regression models (R^2^ = 0.140–0.226), according to this study that examined the drivers of these prejudices among undergraduate medical students.

Based on the subscale’s scoring direction, relational ageism was significantly correlated with affinity for older people (*β* = 0.226, *p* = 0.001); this suggests that lower affinity was associated with greater relational ageism, supporting the protective role of positive attitudes toward older adults in reducing age-based relational concern.

Given the relatively low internal consistency of Internalized Ageism and, to a lesser extent, Relational Ageism (*α* = 0.637) and Internalized Ageism (α = 0.429), respectively, results involving these two subscales should be interpreted cautiously and regarded as preliminary until they are replicated with more reliable measures.

The only significant demographic predictor was younger age, which was linked to higher levels of internalized ableism (*β* = −0.156, *p* = 0.038). However, attitudinal factors showed stronger associations with bias than demographic traits. These results highlight the need for targeted educational interventions, such as structured experiential learning, workshops, and reflective practice, to be applied early in medical training in order to reduce ageism and ableism in healthcare education. To teach future physicians to provide compassionate, inclusive, and equitable treatment for all patients, regardless of age or disability, healthcare curricula must include content on both biases.

## Data Availability

The raw data supporting the conclusions of this article will be made available by the authors, without undue reservation.

## References

[1] Butler RN. Ageism: another form of bigotry. The Gerontologist. (1969) 9:243–6. doi: 10.1093/geront/9.4_Part_1.243, 5366225

[2] Palmore EB. Ageism: Negative and Positive. 2nd ed. New York: Springer Publishing Company (1999).

[3] Campbell FK. Contours of Ableism: The Production of Disability and Abledness. Basingstoke: Palgrave Macmillan (2009).

[4] Wolbring G. The politics of ableism. Development. (2008) 51:252–8. doi: 10.1057/dev.2008.17

[5] Park S. Defence against ableism: does gender representation facilitate rulemaking for people with disabilities? Public Integrity. (2026):1–18. doi: 10.1080/10999922.2026.2638671

[6] Shane K Slonim AD. Medical gaslighting and its impact on vulnerable populations. J Racial Ethn Health Disparities. (2026) 2026:1–6. doi: 10.1007/S40615-026-02850-1, 41639399

[7] Jain A Sadik E Cardenas-Rojas A Mahesh K Ooi P Grami V . Chronic pain in elderly patients: pathophysiology, pharmacologic and non-pharmacologic therapies, and interventional management. Clinic Pharmacol. (2026) 18:506172. doi: 10.2147/CPAA.S506172, 41978902 PMC13070327

[8] Das S Ayalon L. Ageism, ableism, and their intersection: evidence from the longitudinal ageing study in India wave 1. Int Psychogeriatr. (2025):100104. doi: 10.1016/J.INPSYC.2025.100104, 40541451

[9] Folorunsho S. The role of social determinants of health in shaping racial and disability disparities among older adults in the United States. J Aging Soc Policy. (2025):1–17. doi: 10.1080/08959420.2025.2528584, 40605322

[10] Hailu GN Gebru HB Hagos GG Weldemariam AH Tadesse DB Mebrahtom G. The role of family caregivers in supporting older adults in Africa: systematic review. BMC Geriatr. (2025) 25:491. doi: 10.1186/S12877-025-06154-7, 40610950 PMC12231671

[11] Newton C. What I bring to the room: a reflective practice for addressing implicit bias in trainees and practitioners. Reflective Pract. (2025) 27:287–99. doi: 10.1080/14623943.2025.2494328, 37339054

[12] Kim J. Healthcare professionals’ perceptions and experiences of ageism: a qualitative study. Int J Environ Res Public Health. (2025) 22:350. doi: 10.3390/IJERPH22030350, 40238402 PMC11941900

[13] Mohacsi L Stange L Hummers E Kleinert E. Perceptions of age and aging in the context of medical decision-making. A qualitative, case-vignette-based focus group study with physicians, nursing staff, and seniors. SSM - qualitative research. Health. (2026) 9:100703. doi: 10.1016/J.SSMQR.2026.100703, 38826717

[14] Rossi E Di LF Congedo R D’Accolti D. Geriatric transition care between acute hospital and residential healthcare settings: scoping review of current models and proposed conceptual framework. Arch Gerontol Geriatrics. (2026) 143:106160. doi: 10.1016/J.ARCHGER.2026.106160, 41650837

[15] Kovner CT Mezey M Harrington C. Who cares for older adults? Workforce implications of an aging society. Health Aff. (2002) 21:78–89. doi: 10.1377/hlthaff.21.5.78, 12224911

[16] Sivasamy V Ng RQM Lee JHM Mamun K. A narrative review of the role of geriatric advanced practice nurses in the care of frail older adults in Singapore. Geriatrics Nurs (Minheap). (2025) 61:342–8. doi: 10.1016/J.GERINURSE.2024.11.004, 39579452

[17] Hammad BM Salameh B Eqtait FA Maysa K Fashafsheh IH Ayed AJ . Nursing students’ knowledge, attitudes, and behaviors toward aging and ageism in Palestine. BMC Geriatr. (2025) 25:296. doi: 10.1186/S12877-025-05946-1, 40307722 PMC12042619

[18] Huth RM Neurauter VB Millar DC Santoro MC Price J. Empathy education to promote age-friendly healthcare: a scoping review. Physical Occup Therapy Geriatrics. (2025) 43:255–69. doi: 10.1080/02703181.2024.2442310

[19] Miranzadeh F Sefidkar R Rezaeipandari H. Effectiveness of an educational intervention in modifying ageism among nurses in caring for older adults: a randomized controlled trial. BMC Nurs. (2025) 24:1132. doi: 10.1186/S12912-025-03798-Z, 40890733 PMC12400758

[20] Ackerman K Sebat E Draughons Moret JE. Integrative curriculum assessment for inclusion, representation, and equity (I-CAIRE). MedEdPORTAL. (2025) 21:11501. doi: 10.15766/MEP_2374-8265.11501, 40026443 PMC11868286

[21] Park M Chung S. Perceived age stigma and emotional isolation in later life: a sequential mediation through self-esteem and aging anxiety. Aging Ment Health. (2026) 30:336–45. doi: 10.1080/13607863.2025.2543512, 40773687

[22] Huang X Pei X Jian W Xu M. Disability-related loss in lifespan and specific social determinants of health among middle-aged and older adults with disabilities: evidence from China’s aging population. J Aging Soc Policy. (2026) 38:315–33. doi: 10.1080/08959420.2025.2523134, 40581934

[23] Gendron T Camp A Amateau G Mullen M Jacobs K Inker J . The next critical turn for ageism research: the intersections of ageism and ableism. The Gerontologist. (2024) 64:gnad062. doi: 10.1093/geront/gnad062, 37267455

[24] Salleh KM Sulaiman NL Gloeckner G Ramli R Al-Asfour A. Analysing Likert scale type data and interpretation in TVET research: a guideline for the novice researcher. Architecture Image Stud. (2026) 7:550–60. doi: 10.62754/AIS.V7I1.871

[25] Işik A Daşbaş S Şatıroğlu GP. Older adults’ views about ageism in Turkey. Educ Gerontol. (2026):1–21. doi: 10.1080/03601277.2026.2647766

[26] Williams C Gendron T. A gerontology theory–informed pilot intervention to enhance geriatric physical therapy education. Gerontol Geriatr Educ. (2025) 47:399–409. doi: 10.1080/02701960.2025.2573367, 41084155

[27] Perrin PB Christ BR Vargas TA Dini ME Ertman B Cull SL . The internalized ableism inventory: scale development using a hybrid artificial intelligence and community-based participatory research design. Rehabil Psychol. (2025) 71:370–82. doi: 10.1037/REP0000639, 40991817 PMC12462894

[28] Lindsay S Phonepraseuth J Leo S Abdeahad N. The impact of ableism on the health and well-being of children and youth with disabilities: a systematic review. Disabil Rehabil. (2026) 48:2265–87. doi: 10.1080/09638288.2025.2578425, 41157898

[29] Steward A Lee Y Keane CTF Cho YI. Development of the “aging together” anti-ageism peer support intervention for older adults. Clin Soc Work J. (2025) 2025:1–10. doi: 10.1007/S10615-025-01010-Y, 30311153

[30] Drljić K Lebeničnik M. Adaptation of the Slovenian version of the symbolic ableism scale. Cogent. Education. (2025) 12:2461038. doi: 10.1080/2331186X.2025.2461038, 37339054

[31] Levy BR. Stereotype embodiment: a psychosocial approach to aging. Curr Dir Psychol Sci. (2009) 18:332–6. doi: 10.1111/j.1467-8721.2009.01662.x, 20802838 PMC2927354

[32] Pettigrew TF Tropp LR. A meta-analytic test of intergroup contact theory. J Pers Soc Psychol. (2006) 90:751–83. doi: 10.1037/0022-3514.90.5.751, 16737372

[33] Gheorghiu A Goldschmidt E. Socio-educational approaches for vulnerable groups: rural children and older adults. (2026) 40:514–538. Available online at: https://www.uav.ro/jour/index.php/jpe/article/view/2628 (Accessed April 22, 2026)

[34] Iudici A Cottone P. Stigma and clinical implications in educational discourses: an ethnopsychology description of special educational needs configurations in Italian school culture. Hum Arenas. (2026) 2026:1–26. doi: 10.1007/S42087-026-00568-4, 30311153

[35] Kaya Sağlam Ö Avcı Ş. Ageism in healthcare: attitudes of physiotherapists. Physic. Occup. Therapy Geriatrics. (2026):1–18. doi: 10.1080/02703181.2026.2633535

[36] Imperato PJ. Medical education in the United States and reflections on accelerated pathways. J Community Health. (2026) 51:204–10. doi: 10.1007/S10900-026-01558-X, 41793609

[37] Lone A Zareen H Alturkie FM Alnawah AK Hadadi AS Alhedhod AT . A cross-sectional study of career choice in obstetrics and gynaecology and its influencing and discouraging factors among medical students in eastern Saudi Arabia. Front Med (Lausanne). (2026) 13:1795107. doi: 10.3389/FMED.2026.1795107, 41797795 PMC12960527

[38] Alwawi DA Madi H Abu-Dahab SMN AlHeresh R. Beyond learning preferences: exploring the relationship between learning styles and sensory processing among university students. BMC Med Educ. (2026) 26:452. doi: 10.1186/S12909-026-08655-4, 41680784 PMC13005565

[39] Tabatabaei A Nelson E Glaser A Ludwig R Russell J Phadnis MA . Predictors of cognitive behavioural therapy for insomnia in older adults. Behav. Sleep Med. (2026):1–10. doi: 10.1080/15402002.2026.2657913, 41994939

[40] Zhang R Huang M Xie J Yang Y Yan J Wu Z . Efficacy of an integrated case-based learning and team-based learning model within the subjective, objective, assessment, and plan framework in physical therapy education. Front Med (Lausanne). (2026) 13:1767677. doi: 10.3389/FMED.2026.1767677/FULL, 41797770 PMC12960088

[41] Villalba K. Disability and social justice: examining intersectionality and policy-making shortcomings in Colombia. J Hum Rights Soc Work. (2026) 11:408–22. doi: 10.1007/S41134-026-00442-X, 30311153

[42] McKee K McCall V Theakstone D Wilson K Reid L Gilroy R . Understanding the intersectional stigma of ageing, disability, and place: a systematic literature mapping review. Housing Studies. (2026) 41:50–70. doi: 10.1080/02673037.2024.2421844

[43] Langmann E Goldberg DS. Ageism, ableism, and epistemic injustice: tracing absence and construction in bioethics and public health. Am J Bioeth. (2026):1–13. doi: 10.1080/15265161.2026.2637062, 41860240

[44] Cohen J. Statistical Power Analysis. 2nd ed. Erlbaum (1988)., 41860240

[45] Hair JF . Multivariate Data Analysis. 8th ed. Cengage (2019)., 41860240

